# Reduction in child mortality in Ethiopia: analysis of data from demographic and health surveys

**DOI:** 10.7189/jogh.06.020401

**Published:** 2016-12

**Authors:** Tanya Doherty, Sarah Rohde, Donela Besada, Kate Kerber, Samuel Manda, Marian Loveday, Duduzile Nsibande, Emmanuelle Daviaud, Mary Kinney, Wanga Zembe, Natalie Leon, Igor Rudan, Tedbabe Degefie, David Sanders

**Affiliations:** 1 Health Systems Research Unit, South African Medical Research Council, Cape Town, South Africa; 2 Saving Newborn Lives/Save the Children, Cape Town, South Africa; 3 Biostatistics Research Unit, South African Medical Research Council, Pretoria, South Africa; 4 School of Public Health, University of the Western Cape, Cape Town, South Africa; 5 School of Mathematics, Statistics and Computer Science, University of Kwazulu–Natal, Durban, South Africa; 6 Centre for Population Health Sciences and Global Health Academy, University of Edinburgh Medical School, Teviot Place, Edinburgh, Scotland, UK; 7 UNICEF, Ethiopia Country Office, Addis Ababa

## Abstract

**Background:**

To examine changes in under–5 mortality, coverage of child survival interventions and nutritional status of children in Ethiopia between 2000 and 2011. Using the Lives Saved Tool, the impact of changes in coverage of child survival interventions on under–5 lives saved was estimated.

**Methods:**

Estimates of child mortality were generated using three Ethiopia Demographic and Health Surveys undertaken between 2000 and 2011. Coverage indicators for high impact child health interventions were calculated and the Lives Saved Tool (LiST) was used to estimate child lives saved in 2011.

**Results:**

The mortality rate in children younger than 5 years decreased rapidly from 218 child deaths per 1000 live births (95% confidence interval 183 to 252) in the period 1987–1991 to 88 child deaths per 1000 live births in the period 2007–2011 (78 to 98). The prevalence of moderate or severe stunting in children aged 6–35 months also declined significantly. Improvements in the coverage of interventions relevant to child survival in rural areas of Ethiopia between 2000 and 2011 were found for tetanus toxoid, DPT3 and measles vaccination, oral rehydration solution (ORS) and care–seeking for suspected pneumonia. The LiST analysis estimates that there were 60 700 child deaths averted in 2011, primarily attributable to decreases in wasting rates (18%), stunting rates (13%) and water, sanitation and hygiene (WASH) interventions (13%).

**Conclusions:**

Improvements in the nutritional status of children and increases in coverage of high impact interventions most notably WASH and ORS have contributed to the decline in under–5 mortality in Ethiopia. These proximal determinants however do not fully explain the mortality reduction which is plausibly also due to the synergistic effect of major child health and nutrition policies and delivery strategies.

Ethiopia has achieved remarkable declines in under–5 mortality. According to the 2015 UN Inter–Agency Group for Child Mortality Estimation (IGME) report, Ethiopia reached its target for Millennium Development Goal 4 for child survival with an estimated under–five mortality rate of 59 per 1000 live births in 2015, a decline from 205 in 1990. This represents an average reduction in mortality of 5% per year; higher than the average for sub–Saharan Africa (2.9%) [1].

Major policy and program activities related to child survival were initiated in Ethiopia between 2003 and 2013 which built on major reforms starting from the 1990s to decentralise and reorganise the health system. An ambitious Health Extension Programme (HEP) was launched in 2003 which aimed to provide universal access to mainly preventive primary health care services [2,3], through more than 34 000 locally recruited, government–salaried mostly female health extension workers (HEWs) who receive one year of training. Two HEWs have been placed in each health post to serve a kebele, the smallest administrative unit of about 5000 people. HEWs split their time between outreach activities and their health post. Outreach activities include: conducting household visits, organizing communities to participate in the expansion of HEP services, educating families to adopt healthy life–styles and serve as ‘model families’ in their neighborhood. HEWs focus on delivering 16 primary health care (PHC) packages of services including family health promotion, communicable disease prevention and control, hygiene and environmental health and health education and communication services. More recently in 2011, a network of volunteers (Health Development Army), drawn from “model family” households, support the HEWs by providing essential health messages to the community [3,4].

The launch of the HEP in 2003 was followed by the Health Sector Development Programme and the National Child Survival Strategy in 2005. At around the same time there was national scale up of community–based treatment of severe acute malnutrition using ready–to–use therapeutic food [5]. From 2006, when the HEP was fully operational, until the end of 2009, HEWs were involved mainly in preventive and promotive work while their treatment services included the diagnosis and treatment of only malaria, diarrhea (not including low osmolarity ORS) and severe acute malnutrition. A major health policy change occurred in 2009 which enabled HEWs to administer antibiotics (for suspected pneumonia) and zinc (for diarrhea) in the community, while the scale up of integrated community case management (iCCM) only began in 2011.

This paper examines changes in mortality and coverage of child survival interventions in Ethiopia between 2000 and 2011. The impact of changes in coverage of child survival interventions on under–5 lives saved was estimated using the Lives Saved Tool.

## METHODS

### Data sources

We used full birth and death history data collected from women aged 15 to 49 years in nationally representative surveys: namely the 2000 Demographic and Health Survey (DHS) the first DHS to be undertaken in Ethiopia, 2005 DHS and the 2011 DHS to calculate under–5 mortality. The surveys covered 14 072, 13 721, and 16 702 households respectively.

To assess trends in coverage of child survival interventions and nutritional status we used the same three Ethiopian DHS surveys. The surveys provide detailed information about the health and nutritional status of women and children and coverage of health care services. The analysis included all survey data sets available with full data, including sampling weights, to allow for re–analysis (see Table S1 in the **Online Supplementary Document(Online Supplementary Document)** for further details on the surveys). To assess coverage of malaria interventions two separate Malaria Indicator Surveys (MIS) were used since these surveys sample specifically from malaria endemic areas. Malaria is seasonal in most parts of Ethiopia, with variable transmission and prevalence patterns affected by the large diversity in altitude, rainfall, and population movement. The MIS from 2007 [6] and 2011 [7] focus on malarious areas defined as <2000m in altitude mapped by global positioning system (GPS); hence these provide a more appropriate estimate of coverage of malaria interventions than the DHS surveys [7]. All of the surveys provided cross–sectional data on intervention coverage in their respective years; however for the MIS, primary data are not available and only point estimates are presented. Definitions and data sources for all indicators can be found in Table S2 in **Online Supplementary Document(Online Supplementary Document)**.

### Statistical analysis

We used a direct method for estimating under–5 mortality based on the synthetic cohort approach [8,9]. Under this concept, age–specific mortality probabilities for narrow age ranges and defined periods are calculated using death events and exposures. These probabilities are combined to compute the probability that a child has not died before reaching age 5 years [9]. Under–five mortality rates were computed for successive five year periods preceding the 2011 DHS. For the purposes of this analysis, mortality rates were calculated for 5–year periods starting from 1987–1991 up until 2007–2011 (the 5–year period immediately prior to the 2011 DHS). Survival probabilities were calculated over age ranges; 0, 1–2, 3–5, 6–11, 12–23, 24–35, 36–47, 48–59 months as recommended by DHS (Section B in **Online Supplementary Document(Online Supplementary Document)**) [9]. The standard errors for the computed mortality estimates were obtained using the Jackknife variance estimation, a repeated sampling method [8]. A series of mortality estimates were obtained by deleting and replacing each primary sampling unit; this produced a sample of under–5 estimates, from which the variance was computed in turn. We also estimated the average annual change (AAC) in mortality using mortality estimates for the periods 1987–1991 and 2007–2011 (Section B in the **Online Supplementary Document(Online Supplementary Document)**).

We analyzed primary data from three Ethiopia DHS surveys to assess coverage trends for 10 indicators which represent high impact maternal and child health interventions; three additional malaria intervention indicators are presented as point estimates. We re–calculated all coverage indicators using standard indicator definitions [10] for tracking progress toward MDG 4. The sampling design of these DHS surveys, such as clustering at enumeration areas and sampling weights (due to non–proportional sampling), were taken into account. Except for the malaria indicators, coverage estimates for rural areas are presented to reflect the focus of the HEP on universal access. We considered malaria indicators for endemic areas only. The 95% confidence intervals were used to assess whether the changes were significantly different across the three time periods.

We computed anthropometric indicators for stunting (height–for–age) and underweight (weight–for–age) in children younger than three years of age from information on age, height and weight in the surveys applying the WHO child growth standards [11]. Moderate or severe (below minus two standard deviations (SD) from the median) and severe (below minus three standard deviations (SD) from the median) were calculated for both nutritional measures. Infant feeding indicators such as exclusive breastfeeding and micronutrient intake (vitamin A supplementation) were calculated by age of the child. We used Stata (version 13) (Stata Corporation, College Station, Texas, USA) for all mortality and coverage analyses.

We used the Lives Saved Tool (LiST) to estimate the number of deaths averted in 2011 due to changes in coverage since 2000. We compared the changes in mortality produced in LiST with single year estimates from IGME [12] as well as the five–year estimates produced in this analysis using DHS data. LiST uses country–specific or region–specific baseline information on mortality rates and causes of death as well as background variables (fertility, exposure to *Plasmodium falciparum*, stunting rates) and current coverage of more than 60 interventions and their associated effectiveness values [13–16] relative to specific causes of death and risk factors to estimate the deaths averted, overall and by specific interventions. The modeling methods have been widely published including discussion of the limitations [16–18]. We used 2000 as the baseline year and projected forward to 2011 using all available national data on changes in intervention coverage and nutritional status (Section C and Table S5 in the **Online Supplementary Document(Online Supplementary Document)**).

Specific input values used in this LiST application are available in Table S6 in **Online Supplementary Data(Online Supplementary Document)**. The analysis was done with the program Spectrum/Lives Saved Tool, version 5.04 (Johns Hopkins University, Baltimore Maryland, USA).

## RESULTS

**Figure 1:**
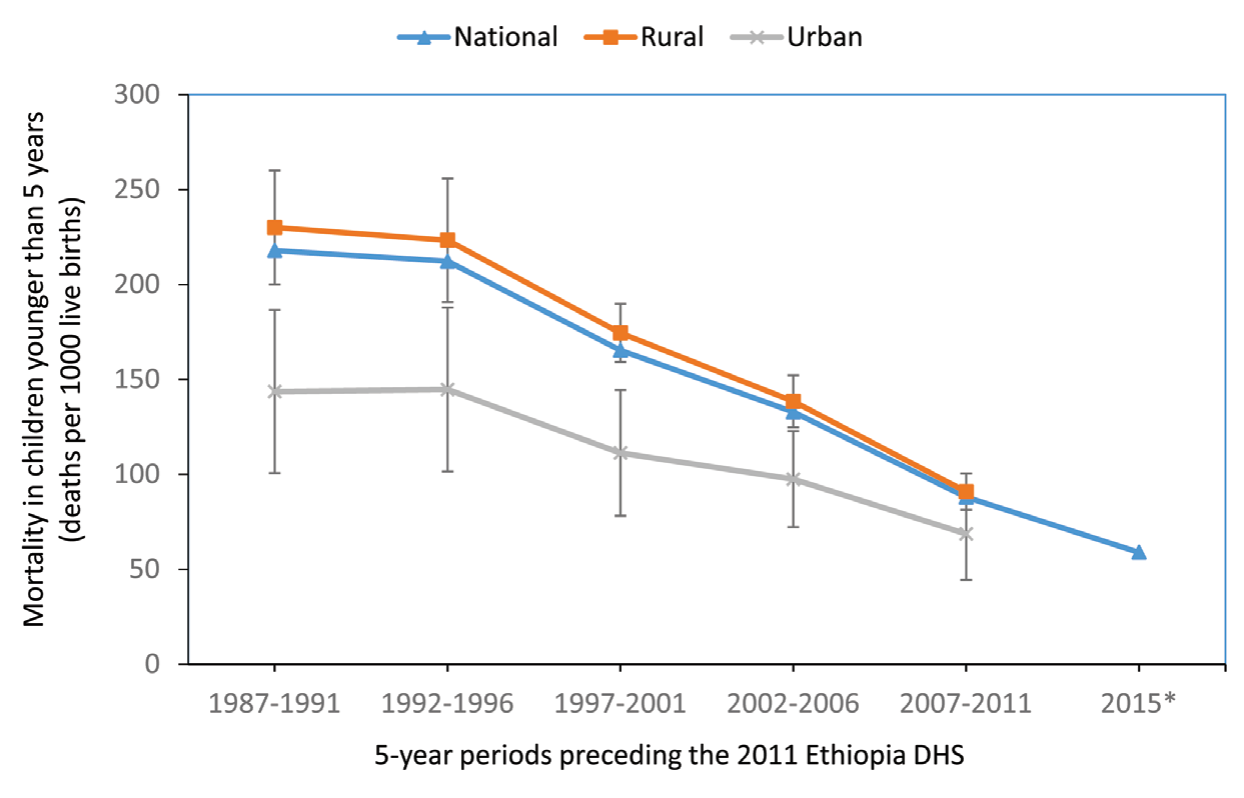
Under–5 mortality rates nationally and in urban and rural areas, Ethiopia, 1987–2011. Data are from analysis of the 2011 national Demographic and Health Survey (DHS) in Ethiopia. Vertical lines show 95% confidence intervals for survival probabilities for the rural and urban estimates. Dates on the x–axis represent the 5–year periods preceding the 2011 Ethiopia DHS. *The 2015 estimate is from the IGME child mortality database (source: UNICEF, [1]).

The national mortality rate in children younger than 5 years decreased rapidly from 218 child deaths per 1000 live births (95% CI 183–252) in the period 1987–1991 to 88 child deaths per 1000 live births in the period 2007–2011 (95% CI 78–98) with an average annual change of –4.5%. The mortality rate was significantly lower in urban areas, compared to rural areas up until the most recent period (2007–2011) where the confidence intervals for the two estimates overlap indicating that the urban mortality estimate was no longer significantly different from the rural estimate (**Figure 1**). Large declines in mortality were also noted in the poorest wealth quintile and among mothers with no education (see Figure S2 and Figure S3 in **Online Supplementary Document(Online Supplementary Document)**).

**Figure 2:**
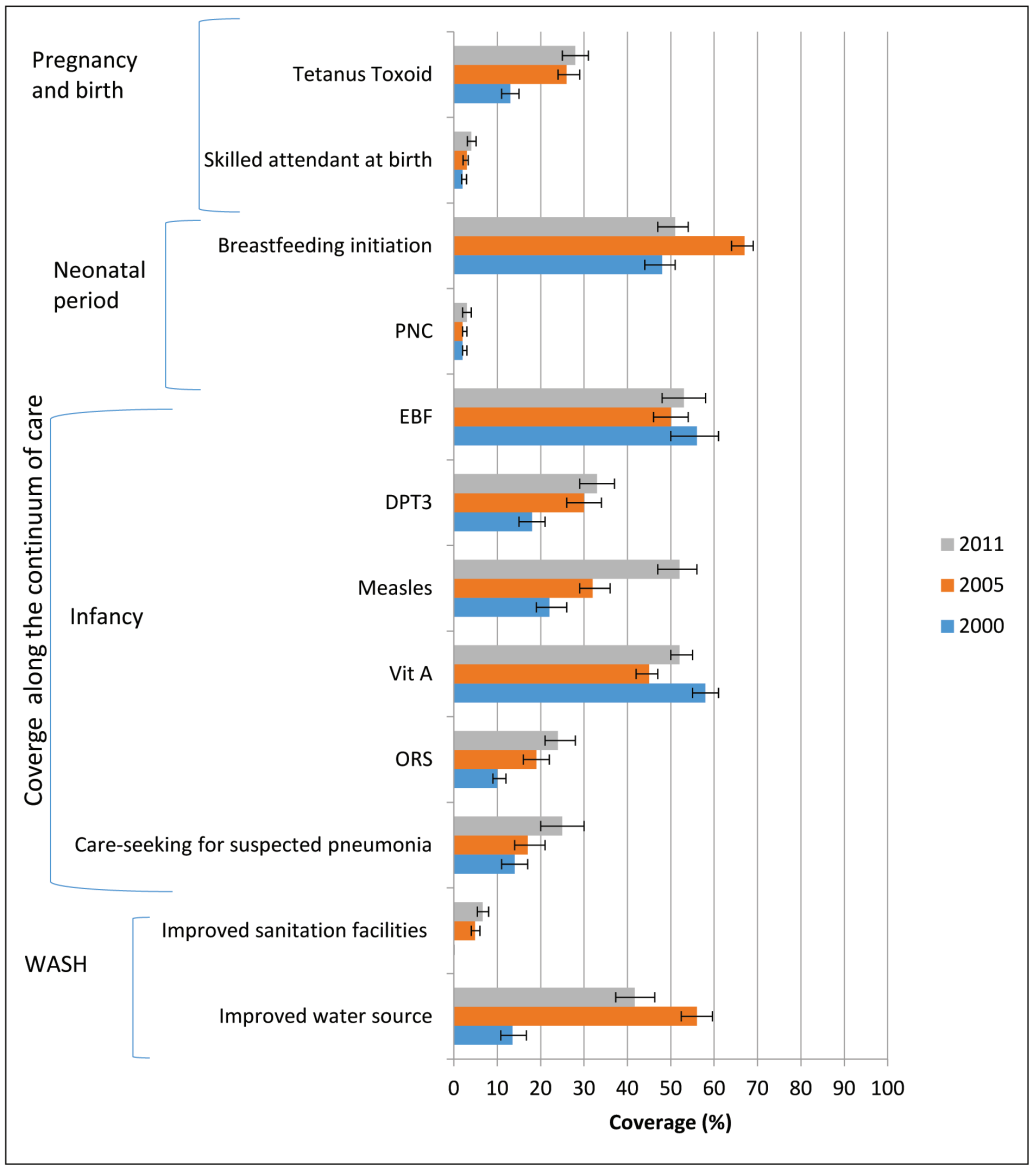
Rural coverage levels for high impact interventions across the continuum of care in Ethiopia as measured in Demographic and Health Surveys (DHS); 2000, 2005 and 2011. Bars represent 95% confidence intervals. DPT3 – three doses of diphtheria, pertussis and tetanus vaccine; ORS – oral rehydration salts; Breastfeeding initiation refers to newborn babies put to the breast within 1 hour of birth; Tetanus Toxoid – percentage of women with a live birth in the last 2 years who received at least 2 doses of tetanus toxoid vaccine during the last pregnancy; PNC – percent of women with live births in the past 2 years who received postnatal care within 2 days after delivery; EBF – exclusive breastfeeding.

Significant improvements in the coverage of interventions relevant to child survival in rural areas of Ethiopia between 2000 and 2011 were noted for all indicators except for vitamin A coverage, breastfeeding initiation, exclusive breastfeeding, skilled attendance at birth and postnatal care (**Figure 2**). Coverage of breastfeeding initiation and exclusive breastfeeding remained high (around 50%) throughout the period of analysis, skilled attendance at birth and postnatal care remained low (<5%) and vitamin A supplementation coverage remained at around 50%. Coverage of improved water source and sanitation, DPT3 and ORS achieved greater gains in the 2000–2005 period while coverage of careseeking for suspected pneumonia and measles vaccination had larger percentage point gains in the 2005 to 2011 period.

**Figure 3:**
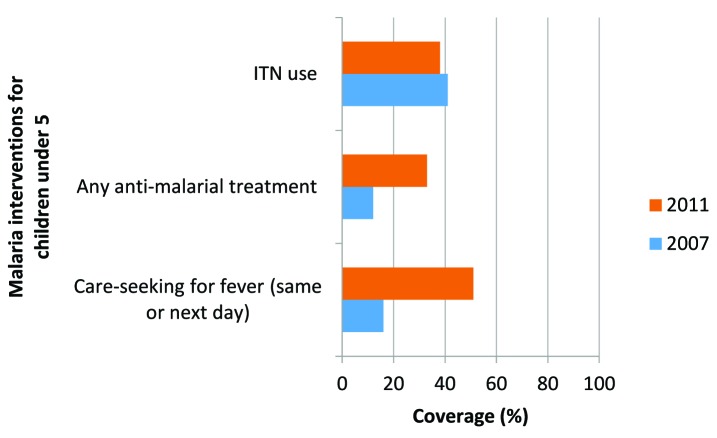
Coverage of malaria interventions in malaria endemic areas of Ethiopia, Malaria Indicator Surveys 2007 and 2011. ITN – insecticide treated nets.

With regard to malaria indicators, increases were noted in timely care–seeking for fever and malaria treatment, the largest being for timely care–seeking for fever rising from 16% to 51%. There was little change in coverage of children under–5 sleeping under insecticide–treated nets (ITNs) (41% to 38%) (**Figure 3**).

**Figure 4:**
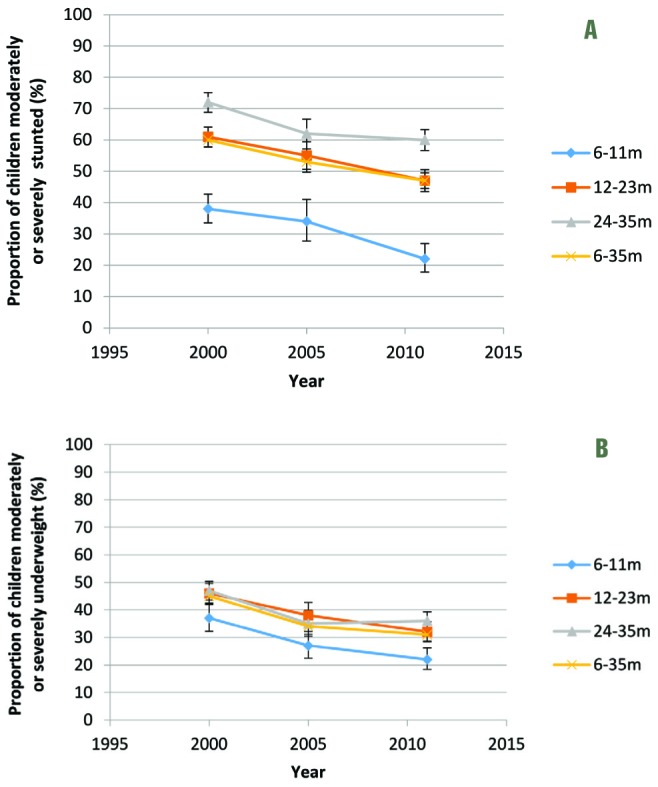
Prevalence of stunting (**A**) and underweight (**B**) by age in children in Ethiopia in the Demographic and Health Surveys (DHS); 2000, 2005 and 2011.

Overall, the prevalence of moderate or severe stunting in children aged 6–35 months declined significantly across both survey periods (2000–2005 and 2005–2011) (**Figure 4**, panel A) with an overall reduction of 13 percentage points (pp). The proportion of children who were moderately or severely underweight also declined significantly among children 6–35 months between 2000 and 2005 (by 11 pp) but did not change significantly between 2005 and 2011. The same trend was seen across all age groups (**Figure 4**, panel B).

Starting at a baseline mortality rate for children younger than 5 years of 146 per 1000 livebirths in 2000 and using available mortality and coverage data up until 2011, LiST predicted under–five mortality to be 119 in 2011, much higher than both the IGME 2011 estimate of 71 (56 to 88) and the 2007–2011 5–year DHS estimate of 88 (78 to 98), and placing it outside the upper confidence range of both estimates.

**Figure 5:**
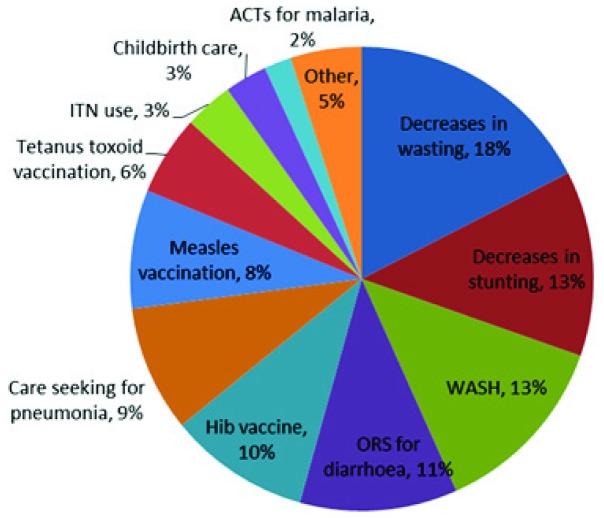
Percentage of child lives saved in 2011 in Ethiopia, by intervention. ACTs – Artemisinin–based combination therapies, ITN – insecticide treated nets, ORS – oral rehydration solution, WASH – water, sanitation and hygiene.

We calculated the proportion of child lives saved in 2011, by intervention or change in nutritional status, using the LiST estimation of 60 700 deaths averted in 2011 (relative to the situation in 2000) as a denominator. The main factors contributing to the prevention of these deaths in 2011 included nutritional interventions resulting in decreases in wasting rates (18%, 11 400 deaths averted) and stunting rates (13%, 8400 deaths averted), water, sanitation and hygiene (WASH) interventions (13%, 8300 deaths averted), ORS for diarrhea (11%, 7200), and the introduction of the Hib vaccine (10%, 6400 deaths averted) (**Figure 5**). Decreases in breastfeeding rates between 2005 and 2011 resulted in an additional 2300 deaths.

## DISCUSSION

Ethiopia has achieved a remarkable decline in under–5 mortality which has occurred in both rural and urban areas and among the poorest and least educated mothers. Our analysis of rural coverage for child survival interventions shows significant change between 2000 and 2011 for several high impact interventions including measles and DPT immunization, ORS coverage, access to an improved water source and care–seeking for suspected pneumonia. For several indicators the biggest coverage change occurred between 2000 and 2005 (tetanus toxoid, DPT3, improved water source and sanitation facilities and ORS) possibly reflecting early impact from the HEP, initiated two years prior to the 2005 DHS, particularly elements such as outreach services, greater access to curative care at health post level, the multi–sectoral approach and a focus on prevention and promotion through model families. Coverage of care–seeking for suspected pneumonia and coverage of ORS treatment for diarrhea was still low in 2011 (both around 25%) although this represents a significant increase from very low coverage levels (around 10%) for both indicators in 2000.

With respect to nutritional status of children, we report a significant decline in both stunting and underweight nationally and our LiST analysis has found that a total of 31% of deaths averted were estimated to be due to decreases in stunting and wasting rates. These shifts in nutritional status of children do not appear to be driven by improvements in breastfeeding practices as exclusive breastfeeding remained high across the period 2000–2011 at around 50% of infants 0–6 months. The changes could plausibly be due to major policy shifts in nutrition which occurred in the country between 2004 and 2008 with the scale up of community management of acute malnutrition at health post level and the development of a national nutrition strategy and program [5,19]. An impact evaluation of the community–based nutrition program in four regions, delivered by HEWs and community volunteers, found substantial changes in infant and young child feeding (increased exclusive breastfeeding) and reductions in stunting prevalence [20]. Furthermore, a recent ecological analysis of patterns in stunting and coverage of nutritional programmes concluded that between 2005 and 2011 the scale up of national nutritional programmes could plausibly have led to reductions in stunting [21].

It is difficult to disentangle the mechanisms whereby socio–economic change and improvements in health coverage interact to generate mortality reduction as these mechanisms can be either direct or indirect and take place concurrently [22]. There are a number of possible explanations for the discrepancy between the IGME–estimated under–five mortality rate and that estimated through our LiST analysis. First, some high impact interventions lack coverage data and so cannot be included in model. Second, it is likely that other contextual changes had influence, which are not captured in LiST. At an economic level, large changes have occurred in the per capita GDP which tripled since 2000 to US$ 355 in 2011; similarly per capita expenditure on health tripled to reach US$ 17.5 in 2011. Furthermore, Ethiopia has received considerable official development assistance (ODA) for maternal, newborn and child health (MNCH) and has successfully guided partner support toward the health sector development program enabling joint financing to ensure implementation of government policies and plans [23]. The annual MNCH ODA has increased from $105 million in 2003 to US$ 215 million in 2010 [24]. Since 2003, Ethiopia has also received US$ 1.4 billion from the Global Fund, 64% of which was spent on HIV/AIDS and between 2004 and 2011 Ethiopia received US$ 1.78 billion from the United States President’s Emergency Plan for AIDS Relief (PEPFAR) [25]. As a result, external resources for health, as a percentage of total health expenditure, increased from 16% in 2000 to 52% in 2011 [26]. This massive funding input could plausibly have had spillover effects on wider health system strengthening beyond the actual programmes it targeted [27,28].

In addition to meeting the MDG 4 target, Ethiopia has also met four other MDG targets including MDG 1 (poverty and hunger), MDG 6 (HIV, malaria and other diseases) and MDG 7 (environmental sustainability). Furthermore at the end of 2015 the country was “on track” to meet MDGs 2 (universal primary education), 3 (gender equality and empowering women) and 5 (maternal health) and was only “off track” on one out of the eight goals (stabilizing debt) [29]. Evidence is emerging that progress made across these multiple sectors which address crucial health determinants has contributed to the fast–track progress in reducing maternal and child mortality in Ethiopia [23,29,30]. This progress does come with some cautionary optimism given the increasing reliance on external resources for health. Other authors have noted this as a challenge facing Sub–Saharan African countries in the post–MDG era. English et al. [31] note that official development assistance (ODA) for health per capita/y in the WHO African Region increased from US$ 2.7 in 2002 to US$ 9.8 in 2010 and while governments’ spending on health has increased, only 6/46 countries in sub–Saharan Africa have met their Abuja target of 15% of their expenditure on health [31].

Several indicators, particularly related to maternal and newborn intervention coverage, showed no improvement in rural areas over the period under analysis. Rural skilled attendance at birth and postnatal care coverage were 4% and 3% respectively in 2011. An analysis of neonatal mortality in Ethiopia found an annual rate of decline of 1.9% between 1995 and 2010, which was even lower (0.9%) for early neonatal mortality (death occurring before 7 completed days of life) the period in which 74% of neonatal deaths occurred [32]. A recently completed 2014 mini–DHS reveals some improvement in these indicators which have reached 9% and 7% respectively, in rural areas in 2014 [33]. Improvement is also seen in another important maternal indicator namely the total fertility rate which has declined from 5.5 in 2011 to 4.5 in 2014 in rural areas. These recent improvements in maternal indicators together with the 2013 launch of community–based newborn care [34] (including sepsis treatment) will hopefully enable the mortality reductions to continue with accelerated progress in reducing newborn deaths.

The endline data for this assessment, the 2011 Ethiopia DHS, occurred at the time of national scale up of the iCCM program and thus provides a picture of coverage and child survival in the absence of an established community–based treatment platform. It is recommended that a comparable analysis be undertaken following the next full DHS to establish the impact of the community delivery platform on child survival and health.

### Strengths and weaknesses of this study

A strength of this study is the re–analysis of primary data to generate mortality and coverage estimates for 10 indicators and nutritional status measures over three time points together with lives saved modeling and a desk review of broader factors hypothesized to impact on child survival.

There are several weaknesses to this analysis. First, primary data were not available for the two MIS to enable us to determine significant changes in malaria interventions; however, for care–seeking and malaria treatment the changes in point estimates are large (>20 percentage points) and the sample sizes for both surveys were over 5000 households, therefore it would be scientifically plausible that these changes are statistically significant. Second, with the LiST analysis, the household survey indicator definitions do not perfectly match LiST indicators in all cases, and some coverage indicators–particularly those related to delivery care–are imputed based on rates of home and facility births. Additionally, the DHS data used in this analysis does not capture some of the interventions included in LiST. These interventions are often high impact for children, eg, therapeutic feeding for severe wasting, and might have changed during the period under consideration. 

### CONCLUSIONS

The collective effect of several positive changes in child nutritional status, and increased coverage of high impact interventions including WASH and ORS have contributed to the decline in under–5 mortality in Ethiopia. These proximal determinants however do not fully explain the mortality reduction which is plausibly also due to the synergistic effect of major child health and nutrition policies and decentralized delivery strategies. Ethiopia’s progress confirms the importance of an integrated approach to child survival [29] and the post MDG era provides an opportunity, through the sustainable development goals, which are comprehensive in addressing specific health interventions as well as key social determinants, for Ethiopia to continue to close gaps related to the social determinants of health. Building on this success will require continued investments and support for universal health coverage with greater attention to maternal and newborn care.
