# Supplementary material for: Reduction in child mortality in Ethiopia: analysis of data from demographic and health surveys
Source: J Glob Health. 2016 Aug 29;6(2):020401. doi: 10.7189/jogh.06.020401 (PMC4854592; doi:10.7189/jogh.06.020401)
Supplement: Online Supplementary Document [file jogh-06-020401-s001.pdf]

Online Supplementary Document

Doherty et al. Reduction in child mortality in Ethiopia: analysis of data from demographic and health surveys

J Glob Health 2016;6:020401

Table of contents

A. Additional details for the methods section 2
B. Mortality analysis 4
C. Additional details regarding the LiST analysis 8

A. Additional details for the methods section

Table S1 provides additional details on the Demographic and Health Surveys (DHS) used in the analysis of mortality and intervention coverage.

Table S1: Description of data for mortality and coverage analysis

Survey and year	Main objectives of the survey	Sampling method	Sample sizes	Sample coverage	Dates of interview	Component of the questionnaire
DHS						
2000	Estimate socio-economic, demographic and health indicators at national and regional levels	Systematic sampling with probabilities proportional to size	539 enumeration areas, 14072 households, 15367 women 15-49	National sample	February - May 2000	Household questionnaire; women 15-49
2005	Estimate socio-economic, demographic and health indicators at national and regional levels	Stratified 2-stage cluster sampling; systematic sampling of clusters; systematic sampling of households	540 clusters, 13721 households, 14070 women 15-49	National sample	April - August 2005	Household questionnaire; women 15-49
2011	Estimate socio-economic, demographic and health indicators at national and regional levels	Stratified 2-stage cluster sampling; systematic sampling of clusters; systematic sampling of households	624 enumeration areas, 16702 households, 16515 women 15-49	National sample	December 2010 to June 2011	Household questionnaire; women 15-49

Table S2 provides definitions and data sources for all indicators included in the coverage trend analysis

Table S2: Indicators included in the coverage analysis

Packages	Coverage indicators	Indicator definition	Data source
**Antenatal care**	Tetanus toxoid vaccination	Proportion of women with a live birth in the last 2 years who received at least 2 doses of tetanus toxoid vaccine during the last pregnancy	Ethiopia Demographic and Household Surveys
**Childbirth**	Skilled attendant at birth	Proportion of births attended by skilled health personnel (doctor, nurse, midwife, auxiliary midwife)	Ethiopia Demographic and Household Surveys
**Breastfeeding**	Breastfeeding initiation	Proportion of newborns put to the breast within one hour of birth	Ethiopia Demographic and Household Surveys
	Exclusive breastfeeding prevalence (0-5 months)	Proportion of infants aged 0-5 months of age who are exclusively breastfed	Ethiopia Demographic and Household Surveys
**Preventive care**	Preventive postnatal care	Proportion of mothers who received a postnatal care visit within two days of birth	Ethiopia Demographic and Household Surveys
	Vitamin A supplementation	Proportion of children 6-59 months who received at least one high dose Vitamin A supplement in the last 6 months	Ethiopia Demographic and Household Surveys
	Improved water source	Proportion of the population using improved drinking water sources	Ethiopia Demographic and Household Surveys
	Improved sanitation facilities	Proportion of homes with access to an improved latrine or flush toilet	Ethiopia Demographic and Household Surveys
	ITN use	Proportion of children under 5 years of age sleeping under an insecticide treated net the previous night	Ethiopia Malaria Indicator Surveys
**Vaccines**	DPT3	Proportion of children 12-23 months of age who received the third dose of DPT or Pentavalent vaccine	Ethiopia Demographic and Household Surveys
	Measles	Proportion of children 12-23 months of age who received measles vaccine	Ethiopia Demographic and Household Surveys
**Curative care**	Diarrhoea	Proportion of children under 5 years of age with diarrhoea in the last 2 weeks who received ORS	Ethiopia Demographic and Household Surveys
	Suspected Pneumonia	Proportion of children under 5 years of age with ARI symptoms in the last 2 weeks whose mothers/caregivers sought care	Ethiopia Demographic and Household Surveys
	Malaria	Proportion of children under 5 years of age with fever in the last 2 weeks whose mothers/caregivers sought care on the same or next day	Ethiopia Malaria Indicator Surveys
		Proportion of children under 5 years of age with fever in the last 2 weeks who received any antimalarial treatment	Ethiopia Malaria Indicator Surveys

B. Mortality analysis

Method of computation of under-five mortality rates

Under-five mortality rates were computed for successive five year periods preceding the 2000, 2005 and 2011 DHS using direct methods based on complete birth histories as recommended by the DHS statistical guide[1]. For each five-year period, age-specific mortality probabilities were computed for eight age groups: 0, 1-2, 3-5, 6-11, 12-23, 24-35, 36-47, 48-59 month using a synthetic cohort concept. This approach involves approximations of the number of children who enter an age range during a specific period. The resulting survival probabilities of death for each age interval were chained together to estimate the survival probability at age five, which was then converted into the under-five mortality rate.

The death rates in this paper are calculated using the following formula:

The estimated probability of death in age-interval *I in period j*:

$$\mathbf{Pr(j}\boldsymbol{i}\mathbf{)} = \boldsymbol{dji}/\boldsymbol{nji}$$

where dj_i is the total number of deaths in period j for age-group *i* in 0, 1-2, 3-5, 6-11, 12-23, 24-35, 36-47, 48-59 months and nj_i is the total number of at risk.

The Life Table method then calculates survival probability as the product of 1 minus the conditional probability of death of all age-intervals as follows:

$\boldsymbol{S(ji)} = \prod_{i=0}^{i=48-60}(\mathbf{1} - \mathbf{Pr}(\boldsymbol{ji}))$ for all *i* in 0, 1-2, 3-5, 6-11, 12-23, 24-35, 36-47, 48-59 months.

Therefore Under 5 mortality rate = **(1- *Sj(i)*) x 1000**

The standard errors for the computed under-five mortality were obtained using Jackknife repeated replications procedures.[2]

Calculation of average annual change (AAC) in mortality

The Average Annual Change in mortality between year 1989 and 2009 was calculated on the log scale assuming linear change as per the below formula:

$$\boldsymbol{r} = \left(\boldsymbol{ln}\left(\frac{\boldsymbol{m_{2009}}}{\boldsymbol{m_{1989}}}\right)\right)\Big/(\mathbf{2009} - \mathbf{1989})$$

Tables S3a, b and c below give under-five mortality estimates for 5 year periods preceding the 2011 DHS with 95% confidence intervals for national, urban and rural areas. Data is from the Ethiopia 2011 DHS.

Table S3a: National under-five mortality rates based on 5-year periods preceding the 2011 EDHS, with the associated 95% confidence intervals.

5 year periods prior to the 2011 DHS	Estimate	95% confidence interval	
		LL	UL
1982-1986	227	187	268
1987-1991	218	183	252
1992-1996	212	176	248
1997-2001	165	147	184
2002-2006	133	119	146
2007-2011	88	78	98

Table S3b: Under-five mortality rates in urban areas based on 5-year periods preceding the 2011 EDHS, with the associated 95% confidence intervals.

5 year periods prior to the 2011 DHS	Estimate	95% confidence interval	
		LL	UL
1982-1986	102	35	169
1987-1991	144	101	186
1992-1996	145	101	188
1997-2001	111	78	144
2002-2006	97	72	123
2007-2011	69	44	93

Table S3c: Under-five mortality rates in rural areas based on 5-year periods preceding the 2011 EDHS, with the associated 95% confidence intervals.

5 year periods prior to the 2011 DHS	Estimate	95% confidence interval	
		LL	UL
1982-1986	247	195	299
1987-1991	230	200	259
1992-1996	223	191	256
1997-2001	174	159	189
2002-2006	138	125	152
2007-2011	91	81	100

Figure S1 below shows the trend in under-five mortality (1972-2011) in 5-year periods using data from three national household surveys.

Figure S1: Trend of under-five mortality rate based on 5-year periods preceding three national household surveys: DHS 2000, 2005 and 2011.

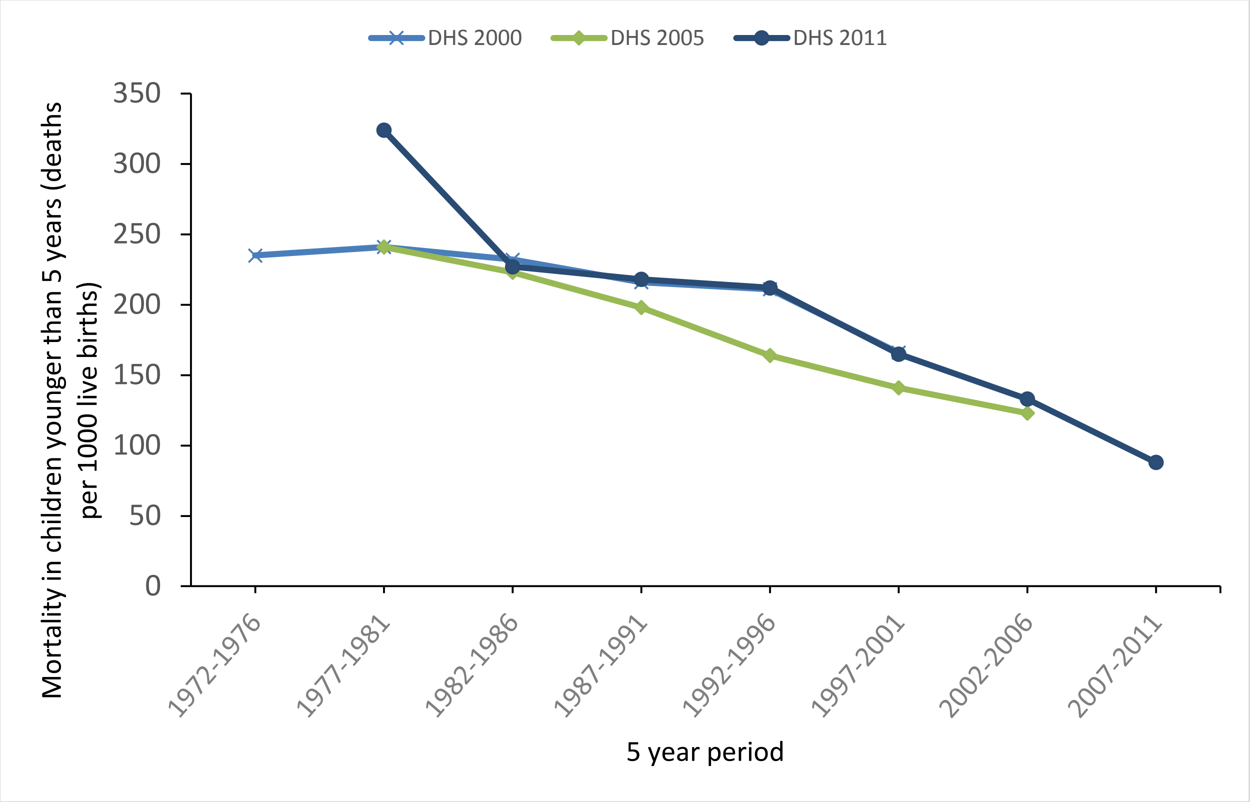

Dates on the x-axis represent 5-year periods preceding the surveys.

Table S4 below provides the under-five mortality rates for 5-year periods preceding three Ethiopia national household surveys.

Table S4: Trends in national under-five mortality rate and sample size from DHS 2000, 2005 and 2011, Ethiopia

	U5MR (per 1000 live births)		
**5 year periods prior to the surveys**	**DHS 2000**	**DHS 2005**	**DHS 2011**
1972-1976	235		
1977-1981	241	241	324
1982-1986	232	223	227
1987-1991	216	198	218
1992-1996	211	164	212
1997-2001	166	141	165
2002-2006		123	133
2007-2011			88
Sample size (household)	14072	13721	16702

Figure S2: Under-5 mortality rates in the poorest and richest wealth quintiles, Ethiopia, 1996-2011

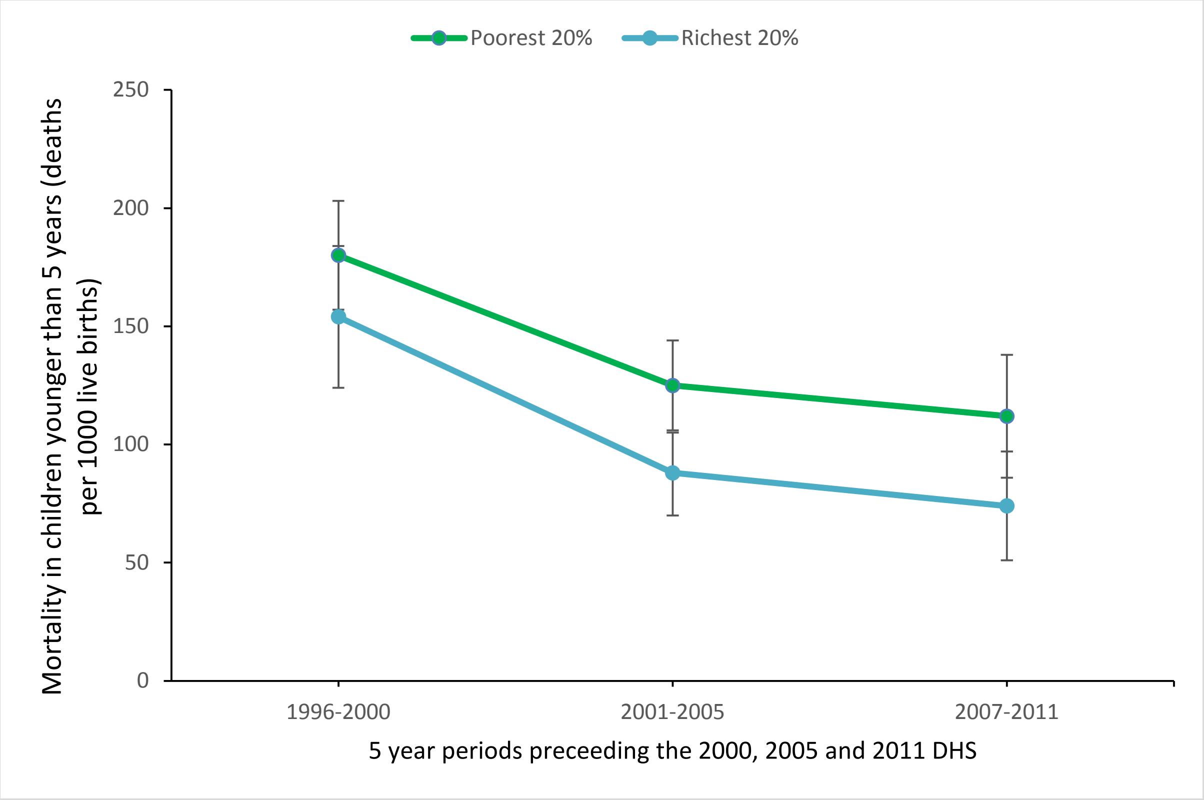

Figure S3: Under-5 mortality rates by education level of the mother, Ethiopia, 1996-2011

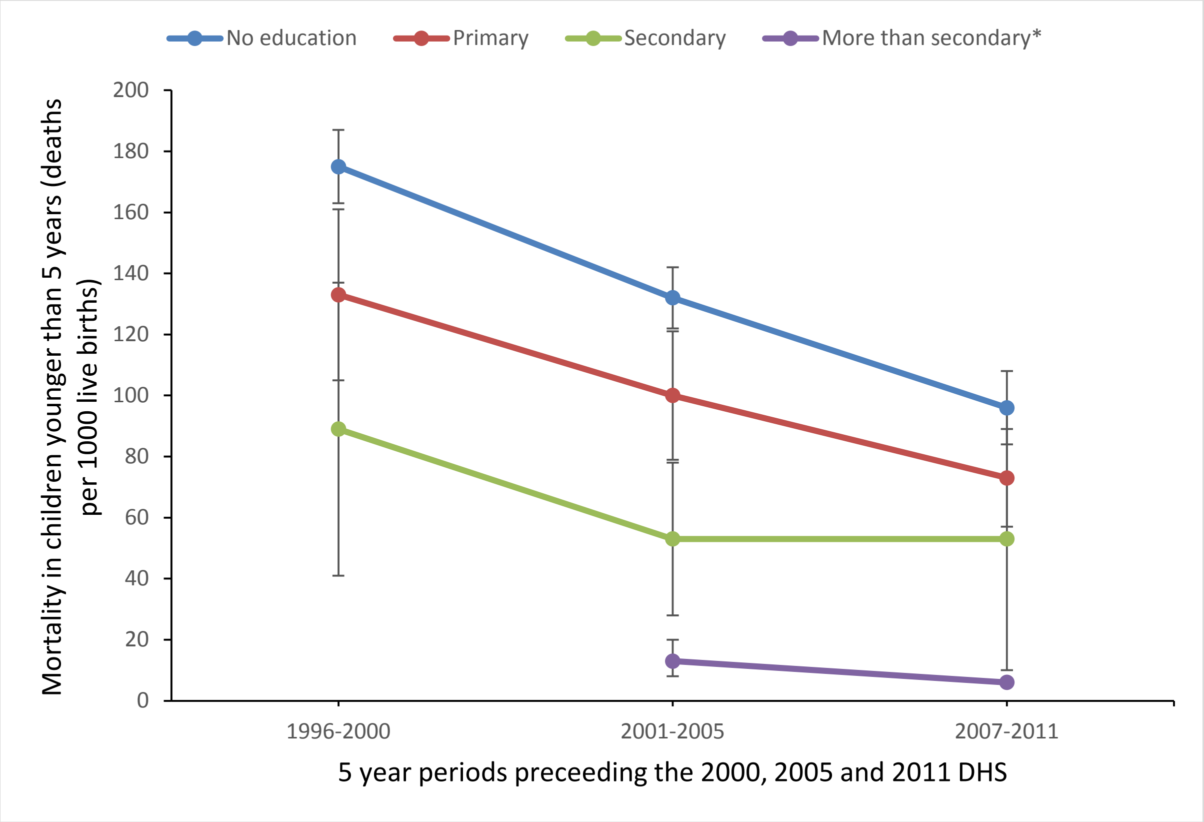

*Sample size too small in 1996-2000 for stable estimate

C. Additional details regarding the LiST analysis

Table S5 shows the data sources used for the baseline characteristics - population, fertility, mortality rates, causes of death and nutrition – for the Ethiopia LiST analyses. Coverage data for key indicators, as well as broader health system indicators, were extracted from all of the available household surveys and other datasets for each time point available and entered into the projections. When coverage data for immunizations was not available from national surveys (e.g. for Hib, Hep B, and pneumococcal conjugate vaccine), WHO/UNICEF coverage estimates were used. Data were interpolated between surveys. Table S6 provides coverage inputs and data sources used in the LiST analysis. For interventions within the childbirth package for which coverage estimates are not available from national household surveys, LiST imputes coverage estimates based on facility birth coverage and skilled attendance at birth coverage. A formula based on facility delivery coverage is used to calculate coverage of basic and comprehensive emergency obstetric care, antenatal corticosteroids, antibiotics for preterm premature rupture of membranes, induction of labour lasting 41+ weeks, active management of the third stage of labour, immediate assessment and stimulation, and neonatal resuscitation. A formula based on home delivery coverage is used to calculate clean birth practices at home and neonatal resuscitation at home. These formulae can be found in the LiST Manual.

Table S5: Additional data used to create LiST projections

Indicator	Source
Population	UN estimates*
Total fertility rate	Demographic and Health Surveys
Stunting, wasting rates	Demographic and Health Surveys
Cause of death	UN estimates
Mortality (under-5, infant, neonatal)	UN estimates*

**Source: www.childmortality.org*

Table S6: Coverage data (%) input and data sources used for LiST analysis

	2000	2005	2011	Data source
Pregnancy				
Antenatal care (4+ visits)	10.6	12.3	19.1	DHS
Tetanus toxoid vaccination (protected at birth)	15.2	28.1	30.5	DHS
IPTp for malaria prevention	1.0	16.8*	37.0	2000=DHS, MIS (*=2007)
PMTCT				UNAIDS
Single dose nevirapine	0	0.7	0.7	
Dual ARV	0	0	17.8	
ART started before current pregnancy	0	0	12.1	
ART started during current pregnancy	0	0	1.4	
Childbirth				
Skilled birth attendance	5.6	5.7	10	DHS
Facility birth	5.0	5.3	9.9	DHS
Interventions within the childbirth package:	3.5	3.6	6.3	LiST default calculations based on facility birth coverage
Clean birth practices	2.8	3.0	5.4	
Immediate assessment and stimulation	5.6	5.8	10.0	
Labor and delivery management	0.3	0.4	0.7	
Neonatal resuscitation	1.3	1.5	2.7	
Antenatal corticosteroids for preterm labor	1.3	1.5	2.7	
Antibiotics for pPRoM	1.3	1.5	2.7	
MgSO4 management of eclampsia	1.3	1.5	2.7	
Active management of the third stage of labor	0.1	0.1	0.2	
Induction of labor for pregnancies lasting 41+ weeks	3.5	3.6	6.3	
Breastfeeding				
Exclusive breastfeeding <1 month	78.4	67.7	70.3	DHS
Exclusive breastfeeding 1-5 months	44	41.6	44.3	DHS
Any breastfeeding 6-11 months	98.6	96.6	97.2	DHS
Any breastfeeding 12-23 months	90.1	91.5	89.2	DHS
Preventive				
Preventive postnatal care	2.4	4.6	6.7	DHS
Complementary feeding (6-9m)	42.5	54.5	51.3	DHS
Vitamin A supplementation	59.9	45.8	52.7	DHS
Improved water source	25.3	61.4	53.7	DHS
Water connection in the home	17.5	23.7	34.3	DHS
Improved sanitation - Utilization of latrines or toilets	0.3	6.8	8.3	DHS
Hygienic disposal of children's stools	22.4	43.1	35.8	DHS
Ownership of insecticide treated nets (ITN/LLIN)	3.4	26.8*	55	2000=DHS, MIS (*=2007)
Vaccines				
BCG	45.6	60.4	66.3	DHS
Polio	34.6	44.7	44.3	DHS
DPT	20.7	31.9	36.5	DHS
H. influenzae b	0	0	27.7	DHS
HepB	0	0	27.7	DHS
Pneumococcal	0	0	0	DHS
Rotavirus	0	0	0	DHS

Measles	26.6	34.9	55.7	DHS
Curative				
Case management of severe neonatal infection	2.9	3.0	3.3	LiST default calculations based on facility birth coverage
ORS - oral rehydration solution	13.1	19.9	26.3	DHS
Antibiotics - for treatment of dysentery	10.4	10.2	9.8	LiST default data
Care-seeking for pneumonia in children	15.8	18.7	27	DHS
Vitamin A - for treatment of measles	59.9	45.8	52.7	Vitamin A preventive coverage used as proxy in LiST
Antimalarials - Artemesinin compounds for malaria	0	0	27	MIS
Cotrimoxazole	0	0	25.8	UNAIDS
ART	0	0.6	18.1	UNAIDS

Household surveys considered included DHS 2000, 2005 and 2011, MIS 2007 and 2011 for malaria indicators

References

1. Rutstein S, Rojas G. Guide to DHS Statistics. Calverton: ORC Macro, 2006.
2. Pedersen J, Liu J. Child mortality estimation: appropriate time periods for child mortality estimates from full birth histories. *PLoS Med* 2012; **9**(8): e1001289.
